# Optimization of the Extraction and Stability of Antioxidative Peptides from Mackerel (*Pneumatophorus japonicus*) Protein

**DOI:** 10.1155/2017/6837285

**Published:** 2017-01-17

**Authors:** Xueqin Wang, Huahua Yu, Ronge Xing, Xiaolin Chen, Song Liu, Pengcheng Li

**Affiliations:** Institute of Oceanology, Chinese Academy of Sciences, Qingdao 266071, China

## Abstract

This study optimizes the preparation conditions for mackerel protein hydrolysate (MPH) by response surface methodology (RSM) and investigates the stability of the antioxidant activity of MPHs (<2.5 kDa). The optimal conditions were as follows: enzyme concentration of 1726.85 U/g, pH of 7.00, temperature of 39.55°C, time of 5.5 h, and water/material ratio of 25 : 1, and the maximum DPPH scavenging activity was 79.14%. The MPHs indicated significant cellular antioxidant activity at low concentrations. Furthermore, the temperature and freeze-thaw cycles had little effect on the antioxidative stability while pH had significant effect on the antioxidative stability. In addition, the MPHs were sensitive to the metal ions, such as Fe^2+^, Fe^3+^, Zn^2+^, and Cu^2+^. Notably, when the concentrations of Fe^2+^ and Fe^3+^ were 5 mM, the DPPH scavenging activities were only 1.1% and 0.6%, respectively; furthermore, Cu^2+^ at a 5 mM concentration could completely inhibit the DPPH scavenging activity of MPHs. In contrast, K^+^ and Mg^2+^ had no notable effect on the antioxidant activity of MPHs. These results may provide a scientific basis for the processing and application of MPHs.

## 1. Introduction

In recent years, there has been increasing interest in finding natural antioxidants, because they can protect the human body from free radicals and retard the progress of many chronic diseases [1]. Many plant and animal sources have been found to possess antioxidant activity, such as* Psidium guajava* leaves [2], soybean protein [3], sheep, and pig blood [4]. Marine organisms are receiving more attention because of their special structure and living environment; notably, a number of studies have been conducted using fish protein hydrolysates as antioxidant peptides, like cod, tuna, salmon, and so on [5–7]. Mackerel (*Pneumatophorus japonicus*) is a kind of pelagic fish which has the characteristics of low economic and high productivity, and how to best utilize these positive characteristics of mackerel needs to be addressed. In recent years, there have been many researches on the processing and utilization of mackerel; for example, Donnelly et al. [8] found the cost and benefits of traceability system implementation both electronically and practically in a mackerel supply chain between Japan and Norway; in addition, the research of lipid oxidation and fishy odor development in protein hydrolysates from the muscle of Indian mackerel was reported [9]; Ferraro et al. [10] also discussed the mackerel canning residues; and Sheriff et al. [11] investigated the hydrolysate from backbones of Indian mackerel, revealing that the hydrolysate contained potent antioxidants and exhibited significant reducing power, free radical scavenging activity, and lipid peroxidation inhibition.

Response surface methodology (RSM) is a statistical multifactorial analysis of experimental variables and response, which offers a better understanding of the experimental process [12]. Besides, RSM as an effective statistical model has been widely used in pharmaceutical and functional foods research [13]. In recently years, RSM model has been widely applied to fish protein [14–16] extraction. For example, Shi et al. [17] has optimized processing parameters of horse mackerel (*Trachurus japonicus*) dried in a heat pump dehumidifier under the conditions from RSM model; Wang et al. [18] studied the hydrolysis conditions for the production of iron-binding peptides from mackerel processing byproducts under the experimental process through RSM system. However, there has been little investigation into the optimization of antioxidative peptide extraction from mackerel.

While various antioxidant peptides have been isolated from different fish proteins, most research has focused on the relationship between their structure and function. To the best of our knowledge, little is known about the effects on the antioxidant activity of peptides of processing and storage conditions, such as temperature, light, pH, phenols, and metals [19]. These factors may affect the bioactivity of peptides and limit their application in food field. Furthermore, the amino acid composition of peptide is complex and may be degraded through deamidation, oxidation, hydrolysis, and cyclization reactions during processing and storage, leading to the loss of antioxidant activity [20]. As an example, high temperatures may alter peptide structures, and the target peptide can be degraded into fragments, thereby losing the antioxidant activity [21]. On the other hand, drying of hydrolysates at high temperature may not destroy the biological activity of peptides [22]. Consequently, it is important to study factors that might affect the stability of the antioxidant activity of peptides during processing and storage.

In our prior work, five proteases (trypsin, papain, neutrase, acid protease, and flavourzyme) were used for hydrolysis to select the optimal mackerel protein hydrolysate (MPH), and the results showed that the hydrolysate produced by neutrase (1.0 × 10^5^ U/g) exhibited the highest DPPH scavenging activity (35.9%) and degree of hydrolysate (15.9%). In this article, we would optimize conditions for the extraction of MPH by the single factor experiment and RSM. Furthermore, in our previous study, we examined the antioxidant activity of different molecular weights of MPH and found that the fractions with molecular weight below 2.5 kDa exhibited the strongest antioxidant activity [23]. Thus, this paper also presents cellular antioxidant activity and the stability of antioxidant activity of MPHs (<2.5 kDa).

## 2. Material and Methods

Fresh mackerel (*Pneumatophorus japonicus*), 210–260 g/fish, was purchased from a seafood market in Qingdao, China. Whole fish were transported on ice to reduce histamine producing. Upon arrival, the fish were washed and the flesh (without head, tail, skin, bones, and blood) was collected, sliced, minced, and stored in plastic bags at −20°C until use. Five proteases (trypsin, papain, neutrase, acid protease, and flavourzyme) were provided by Kangbaotai Co. (Hubei, China). 1,1-Diphenyl-2-picrylhydrazyl (DPPH) and 3,5-di-tert-4-butylhydroxytoluene (BHT) were purchased from Sigma Chemical Co. (St. Louis, MO, USA). The ultrafiltration (UF) system and UF membranes with 2500 Da molecular weight cutoffs were purchased from Laungy Co. Ltd. (Shanghai, China). The growth medium and antibiotics for the cell culture experiments were purchased from Hyclone, USA; serum was purchased from Gibco, Australian. All other chemicals and solvents were of analytical grade.

### 2.1. Preparation of Mackerel Protein Hydrolysates

#### 2.1.1. Single Factor Experiments

In this section, the neutrase-treated hydrolysate was chosen as the best candidate [23], and five major factors (enzyme concentration, pH, extraction temperature, extraction time, and water/material ratio) were selected for the single factor experiments. The mackerel muscle was mixed with deionized water at a various of water/material ratio, and the mixtures were adjusted to the required pH with 0.01 mol/L NaOH or HCl and heated in a water bath to the required temperature before the neutrase was added in proper proportion based on its activity, and the hydrolysis reactions were carried out in a shaking incubator. At the end of the hydrolysis period, the mixtures were heated in boiling water for 10 min to inactivate the proteases. Then the hydrolysates were centrifuged at 18000 ×g (4°C) for 30 min and the supernatants or dried powders by freeze dryer were used for DPPH radical scavenging activity. It is worth mentioning that the DPPH radical scavenging assay, which is simple and accurate, has been widely used to evaluate antioxidative properties of compounds as free radical scavengers or hydrogen donors [24–26].

#### 2.1.2. Scavenging Activity on DPPH Radicals

The DPPH radical scavenging activities of the MPH supernatant were determined as described by Chen et al. [27] with slight modifications. Briefly, 1.0 mL of DPPH (0.1 mmol/L) diluted in ethanol was added to 3.0 mL of MPH supernatant. After vigorous shaking, the mixture was left to stand for 30 min and the absorbance was measured at 517 nm. The DPPH radical scavenging activity was calculated as follows: scavenging rate (%) = [1 − (*A*_1_ − *A*_0_)/(*A*_2_ − *A*_0_)] × 100, where *A*_0_ was the absorbance without DPPH, *A*_1_ was the absorbance in the presence of the MPH supernatant, and *A*_2_ was the absorbance of the control (without sample). All experiments were performed in triplicate.

#### 2.1.3. Scavenging Activity on Hydroxyl Radical

Scavenging activity of MPH supernatant on hydroxyl radicals was performed, using method described by You et al. [28], with a few modifications. Briefly, the reaction mixture contained 1.0 mL of phosphate buffer (PBS, 0.15 mol/L, pH 7.4), 1.0 mL of safranin T (1.0 mM), 0.5 mL of EDTA-FeSO_4_ (2.0 mmol/L), and 1.0 mL of MPH supernatant. After sufficient mixing, 1.0 mL of H_2_O_2_ (3%) was added to the mixture. Following incubation at 37°C for 30 min, the absorbance of the mixture was measured at 520 nm. The hydroxyl radical scavenging activity was calculated as scavenging rate (%) = [(*A*_1_ − *A*_0_)/(*A*_2_ − *A*_0_)] × 100, where *A*_1_ was the absorbance of the MPH supernatant, *A*_2_ was the absorbance without H_2_O_2_, and *A*_0_ was the absorbance of the control. Both *A*_0_ and *A*_2_ were the mixtures with sample solution replaced by deionized water. All experiments were performed in triplicate.

#### 2.1.4. Optimization of MPH Preparative Conditions by RSM

On the basis of the single factor experiments, the five independent variables at five levels were employed in a central composite experimental design (CCD). The five independent variables (enzyme concentration, pH, extraction temperature, extraction time, and water/material ratio) were coded as *X*_1_, *X*_2_, *X*_3_, *X*_4_, and *X*_5_, respectively. The ranges and levels of the variables are given in Table 1, and the complete design consisted of 50 combinations including eight replicates of the center points.

The responses obtained from each set of experimental designs were analyzed by multiple regressions to fit the following quadratic polynomial model:

(1) $$\begin{array}{c} Y=\beta_{0}+\overset{k}{\underset{i=1}{\sum }}\beta_{i}X_{i}+\overset{k}{\underset{i=1}{\sum }}\beta_{ii}X_{i}^{2}+\sum \sum_{i<j}\beta_{ij}X_{i}X_{j}, \end{array}$$

where *Y* is the response variable, *β*_0_ is a constant, and *β*_*i*_, *β*_*ii*_, and *β*_*ij*_ are the linear, quadratic, and interaction coefficients, respectively, while *X*_*i*_ and *X*_*j*_ are the coded independent variables [26].

Design-Expert 8.0 (Stat-Ease, Inc., China) was used to analyze and calculate the predicted responses and experimental design for the DPPH scavenging activity. The analysis of variance table was generated, and the effect and regression coefficients of linear, quadratic, and interaction terms were determined. The statistical significance for each term in the polynomial was determined by computing the *F* value at a probability *P* of 0.05. The regression coefficient was used to perform statistical calculations and the generated 3D surface was from the fitted polynomial equation.

### 2.2. Antioxidant Analyses in HepG2 Cells

#### 2.2.1. Cytotoxicity

The inhibition of HepG2 was assessed by the MTT assay described by Chen et al. [29] with a few modifications. The HepG2 cells were seeded into 96-well culture plates (4 × 10^3^–1 × 10^4^/well) and incubated at 37°C in a humidified atmosphere with 5% CO_2_ for 24 h, then the HepG2 cells were incubated with MPHs at different concentrations (0.5, 1, 2.5, 5, 10, 15, and 20 mg/mL and 100 *μ*L) for 24 h, and the cells without the MPHs were used as a negative control. Then, 20 *μ*L of MTT (5 mg/mL) was added to each well and the plates were incubated for 3 h. After the removal of MTT, dimethyl sulfoxide (DMSO) (150 *μ*L/well) was added and shaken for 10 min; then the absorbance was measured on a microplate reader (Bio-Rad, USA) at a wavelength of 490 nm. Measurements were performed 4 times and the inhibition of HepG2 was evaluated.

#### 2.2.2. Cellular Antioxidant Activity

Cells were placed in a 96-well plate (4 × 10^3^–1 × 10^4^/well) and incubated at 37°C in a humidified atmosphere with 5% CO_2_ for 24 h. Then cells were treated with MPHs at different concentrations (0.05, 0.1, 0.25, 0.5, 1, 2, 2.5, and 5 mg/mL and 100 *μ*L) for 24 h, after which the cells were treated with 1000 *μ*M H_2_O_2_ (100 *μ*L) for another 24 h, and the cellular antioxidant activity of MPHs was tested with MTT assay as described above. The cells without the H_2_O_2_ used as a negative control and the measurements were performed 4 times.

### 2.3. Stability of the Antioxidant Activity of MPHs

#### 2.3.1. Effect of Temperature on the Antioxidant Activity of MPHs

To determine the appropriate temperature range for processing and storage, the MPHs (5 mg/mL) was incubated at −4, 20, 36, 60, 80, and 100°C for 2 h, the condition of −4°C was controlled by fridge, and the other temperatures were controlled by water bath. After the specified time, the sample was immediately cooled in iced water, then centrifuged at 18000 ×g (4°C) for 30 min, and subsequently evaluated for DPPH and hydroxyl radical scavenging activities.

#### 2.3.2. Effect of pH on the Antioxidant Activity of MPHs

The pH range selected for the present study was from 2.2 to 9.2. The pH of the MPHs was adjusted using 1 M NaOH or 1 M HCl, and the MPHs (5 mg/mL) was maintained at room temperature for 2 h. After the specified time, each sample was centrifuged and the DPPH and hydroxyl radical scavenging activities were determined.

#### 2.3.3. Effect of the Freeze-Thaw Cycle on the Antioxidant Activity of MPHs

The MPHs (5 mg/mL) was frozen at −80°C for 2 h firstly and then unfrozen with the running water at room temperature, which was defined as the first cycle. The second cycle was repeat frozen and unfrozen and so on. The sample was centrifuged and the DPPH and hydroxyl radical scavenging activities were determined to illustrate the effect of freeze-thaw cycle on the stability of MPHs.

#### 2.3.4. Effect of Metal Ions on the Antioxidant Activity of MPHs

The effect of metal ions on the antioxidant activity of MPHs was studied by the addition of 20 mM solutions of K^+^, Zn^2+^, Ca^2+^, Fe^2+^, Fe^3+^, Mg^2+^, and Cu^2+^ from KCl, ZnCl_2_, CaCl_2_, FeCl_2_, FeCl_3_, MgCl_2_, and CuCl_2_, respectively. Each metal ion was added in appropriate quantities to attain final concentrations of 0.1, 0.5, 1.0, 2.0, 3.0, 4.0, and 5.0 mM, respectively. The mixtures were incubated at room temperature for 2 h, and the DPPH and hydroxyl radical scavenging activities were measured.

### 2.4. Statistical Analysis

All tests were conducted in triplicate. The experimental data were expressed as the mean ± standard error. LSD and Duncan tests were performed to determine the significant differences between samples within a 95% confidence interval, using SPSS 18.0 statistical software (IBM, USA).

## 3. Results and Discussion

### 3.1. Single Factor Experiments

**Figure 1:**
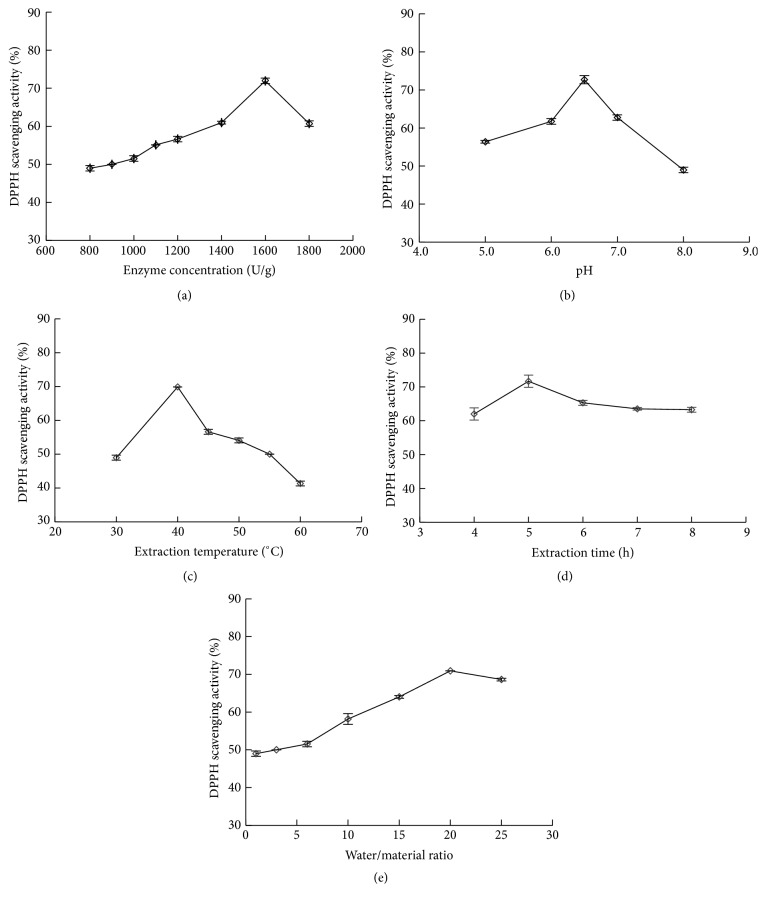
Effects of five single factors on the DPPH scavenging activity.

In this work, the effects of five single factors on the DPPH scavenging activity were investigated (Figure 1), the result showed that, under the range of five single factors, the DPPH scavenging activity increased at first and then decreased; then the optimal conditions are displayed: enzyme concentration of 1600 U/g, pH of 6.5, extraction temperature of 40°C, extraction time of 5.0 h, and water/material ratio of 20 : 1.

### 3.2. Optimization of Extraction Conditions by CCD

According to the single factor experiments, the design matrix and corresponding results obtained from CCD for determining the effects of the five independent variables (*X*_1_, *X*_2_, *X*_3_, *X*_4_, and *X*_5_) were listed in Table 2.

These results showed that the DPPH scavenging activity ranged from 52.97% to 81.62%. The data were analyzed via multiple regression analysis using Design-Expert software to yield the following polynomial equation:

(2) Y=−186.58+0.11X1−11.43X2+7.20X3+1.45X4+2.82X5+2.20E−003X1X2−2.70E−004X1X3+3.88E−003X1X4+1.35E−004X1X5+0.16X2X3+1.22X2X4+0.10X2X5+0.05X3X4+0.06X3X5+0.63X4X5−4.16E−005X12−0.65X22−0.05X32−2.19X42−0.08X52.

Analysis of variance (ANOVA) results for the model were given in Table 3. The corresponding variables were more significant as the *F* value became greater and the *P* value became smaller [30]. It could be seen that the variables with the most significant effects on the DPPH scavenging activity of MPH were certain linear terms (*X*_1_, *X*_2_, *X*_3_, and *X*_5_), quadratic terms (*X*_1_^2^, *X*_3_^2^, and *X*_5_^2^), and interaction terms (*X*_3_ × *X*_5_ and *X*_4_ × *X*_5_). As seen in Table 3, the model showed a good fit with the experimental data, with high values of *R*^2^ (95.56%) and Adj. *R*^2^ (92.49%). The low coefficient value of the variation (CV = 2.56%) clearly suggested a high degree of precision and reliability of the experimental values. This result implied that the hydrolysis process of MPH could be analyzed and predicted by the model.

The effects of variables and their interactions on DPPH scavenging activity were illustrated by 3D response surfaces. The figures displayed the effects of two factors on DPPH scavenging activity while the others were kept at a zero level [31].

**Figure 2:**
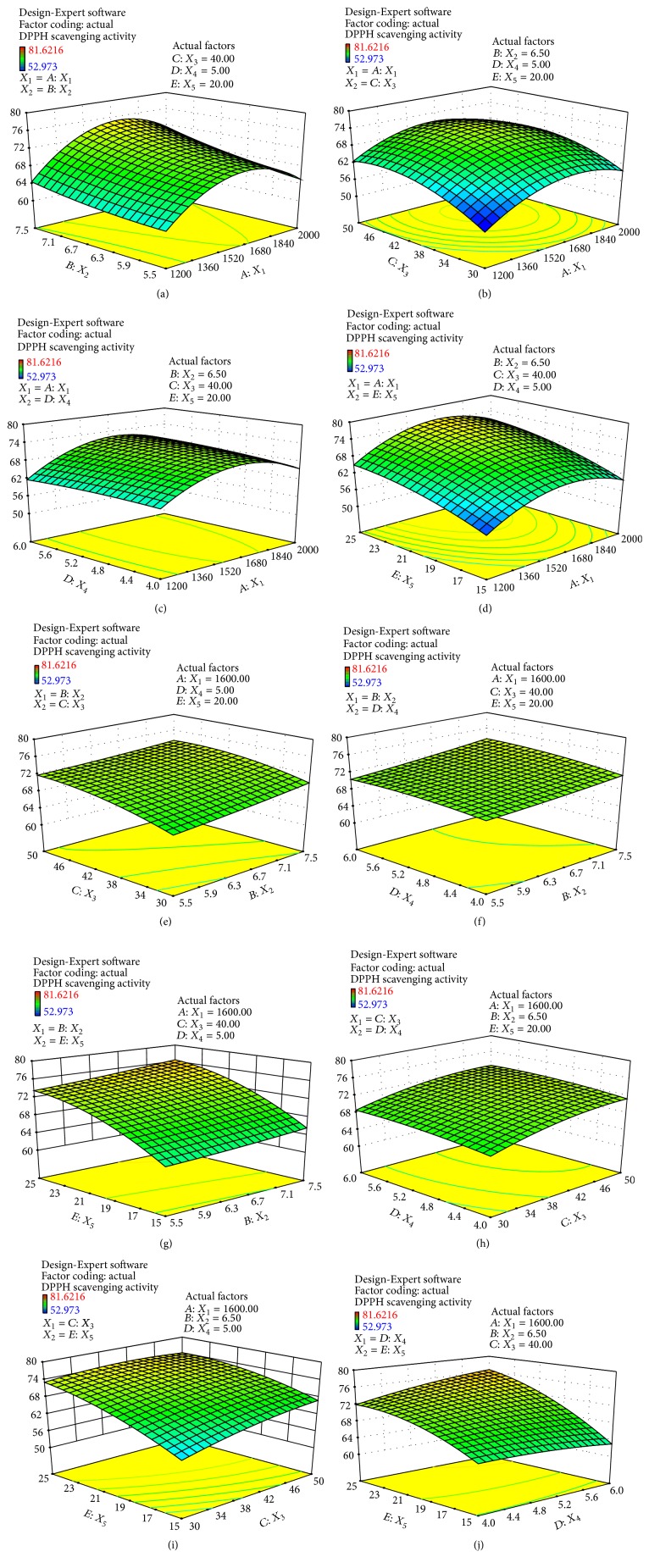
Response surface plots showing the effects of variables (*X*_1_: enzyme concentration; *X*_2_: pH; *X*_3_: extraction temperature; *X*_4_: extraction time; *X*_5_: water/material ratio) on the scavenging activity of MPH (*y*-axis: DPPH scavenging activity).

Figure 2(a) showed that DPPH scavenging activity increased as the enzyme concentration was increased from 1200 to 1600 U/g, but further higher enzyme concentration did not influence the DPPH scavenging activity. The excess enzyme might not participate in the reaction; on the contrary, they increased the concentration of reaction system and restricted the activity of free radical. When the pH increased from 5.5 to 7.5, DPPH scavenging activity had increased slightly, because each protein has different isoelectric point, and then the solubility of protein was affect by pH value.

As shown in Figure 2(b), at lower enzyme concentrations, when temperature increased, DPPH scavenging activity increased slightly; at higher enzyme concentrations, DPPH scavenging activity increased slightly with temperature from 30 to 46°C and decreased slightly with temperature from 46 to 50°C; the maximum DPPH scavenging activity was observed at about 46°C. This was likely because the treatment at approximately 60°C would cause complete denaturation of the peptide [32], and high extraction temperatures may be due to denaturation and inactivation of enzymes and decrease the DPPH scavenging activity. In addition, when enzyme concentrations increased from 1200 to 1800 U/g, DPPH scavenging activity increased significantly; maybe there are more enzymes molecules present in high enzyme concentration; there will be more chances for the hydrolysis to occur [33]; these result is agreed with Fang et al. [34], which found higher DPPH scavenging activity occurring at a high enzyme to substrate ratio. While when enzyme concentration increased from 1800 to 2000 U/g, DPPH scavenging activity decreased slightly, we speculated that too high enzyme concentration may increase the concentration of system and limited the ion activity.

As shown in Figure 2(c), DPPH scavenging activity increased slightly when the extraction time increased from 4 to 5 h and then decreased slightly with an extraction time of 6 h; we inferred that the hydrolysis reaction was powerful in the first four hours and became flat later. As shown in Figure 2(d), when the water/material ratio increased from 10 to 30, DPPH scavenging activity increased significantly; some researchers have studied that increased water added to substrate enhanced enzyme homogeneity and reduced the localized concentration of hydrolysis products [35]. In addition, the DPPH scavenging activity increased firstly and decreased significantly with the enzyme concentrations from 1200 to 2000 U/g; the result was the same as Figure 2(b).

As shown in Figure 2(e), when pH and temperature increased, DPPH scavenging activity increased slightly. As shown in Figure 2(f), with the hydrolysis times increased from 4 to 6 h, DPPH radical scavenging activity increased very slowly when pH increased. In addition, Figure 2(h) showed that DPPH scavenging activity increased slowly with the increase of temperature and extraction time. These results did not conclude that the factors had no effect on DPPH scavenging activity; in other words, we inferred that there was an interaction between the two factors in Figures 2(e), 2(f), and 2(h), because the DPPH scavenging activity was maintained at high value from about 68% to 73%.

Figures 2(g), 2(i), and 2(j) showed that when the water/material ratio increased, DPPH scavenging activity increased significantly, which indicates that a relatively high water/material ratio is desirable to promote the antioxidant activity of hydrolysates.  Figure 2(i) showed that, at lower water/material ratio, DPPH radical scavenging activity increased significantly when temperature increased; at higher water/material ratio, DPPH radical scavenging activity remains unchanged when temperature increased. Some researchers have explained that at lower temperatures, the rate of enzyme heat-inactivation was slower in comparison with the rate of the enzyme catalyzed reaction. At higher temperatures, the increased heat-inactivation rate led to a faster decrease in the number of active catalyst molecules [15]. Furthermore, Figure 2(j) showed that at lower water/material ratio, DPPH radical scavenging activity decreased slightly when hydrolysis times increased; at higher water/material ratio, DPPH radical scavenging activity increased slightly when hydrolysis times increased.

Using Design-Expert 8.0, the optimal hydrolysate conditions were enzyme concentration of 1726.85 U/g, pH of 7.00, temperature of 39.55°C, extraction time of 5.5 h, and water/material ratio of 25 : 1. The maximum DPPH scavenging activity was 79.14%, which was in agreement with the experimental value (79.19%) within a 99% confidence interval, suggesting a good fit between the model and experimental data.

### 3.3. Cytotoxicity Effects

**Figure 3:**
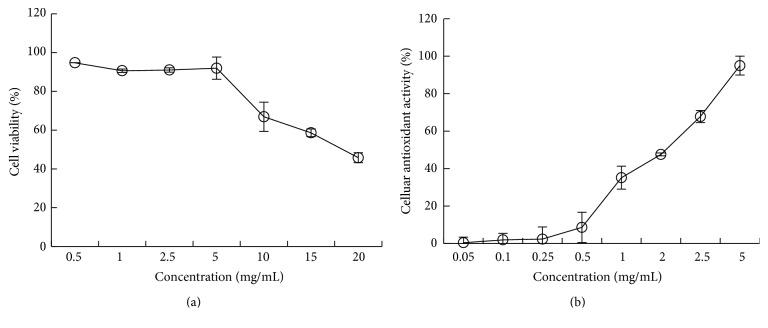
Cellular antioxidant activity of MPHs with various concentrations in HepG2 cells.

Toxicity study was conducted to ascertain that the sample was safe for the proposed treatments on the HepG2 cells. The cellular antioxidant activity of MPHs in HepG2 cells was measured with concentrations of 0.5, 1, 2.5, 5, 10, 15, and 20 mg/mL, respectively. After 24 h of incubation, the effect of MPHs on HepG2 cells viability was analyzed (Figure 3(a)); the results showed that the MPHs were relatively nontoxic to HepG2 cells at concentrations less than 5 mg/mL, with cell viability more than 90%; this was similar to the research by Kong et al. [36]. These preliminary analysis showed that MPHs had low toxicity at high concentrations.  Figure 3(b) showed the cellular antioxidant activity of MPHs which with concentration from 0.05 mg/mL to 5 mg/mL was from 0.41% to 94.95%, and the antioxidant activity of MPHs was significantly dependent on the concentrations.

**Figure 4:**
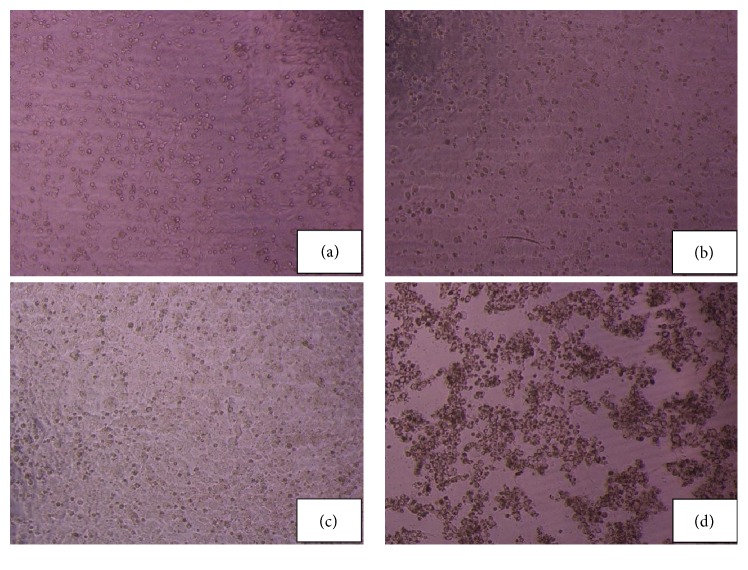
Morphological changes of HepG2 cells in response to untreatment, H_2_O_2_ (1000 *μ*mol/L), MPHs (5 mg/mL) + H_2_O_2_, and MPHs (20 mg/mL) + H_2_O_2_, respectively. The images were captured using a digital camera attached to an inverted microscope.

Figures 4(a)–4(d) showed the image of the cells viewed under a light microscope.  Figure 4(b) showed H_2_O_2_-induced oxidative damage in the untreated HepG2 cells, accompanied by the cell nucleus exposed. After pretreatment with MPHs (5 mg/mL), the HepG2 cells caused significant inhibition of oxidative damage in Figure 4(c), which exhibited viability levels similar to those of the control group in Figure 4(a). However, when the cells were treated with MPHs at the concentration of 20 mg/mL, the cells were seriously damaged, as Figure 4(d) showed that the cell nucleus was exposed, and the cells clustered, indicating that a high concentration of MPHs expressed cytotoxicity. The result showed that the MPHs exhibited significant cellular antioxidant activity within a certain concentration.

### 3.4. Stability of Antioxidant Activity of MPHs

Several protein hydrolysates derived from food protein have become important areas of research for health food [37]. In our prior work, MPHs (<2.5 kDa) exhibited in vitro antioxidant activity and in vivo antifatigue effects [38], indicating that they could be used as natural antioxidants to enhance the antioxidant properties of functional foods. In contrast, only a few studies have examined the stability of peptide bioactivity. Some studies have reported that technological processes used in food manufacture affect the functional, nutritional, and biological properties of food protein [39]. Given these results, it is necessary to study the effect of different factors, such as temperature, pH, freeze-thaw frequency, UV, and metal ions on the antioxidant activity of MPHs.

#### 3.4.1. Effect of Temperature on the Antioxidant Activity of MPHs

**Figure 5:**
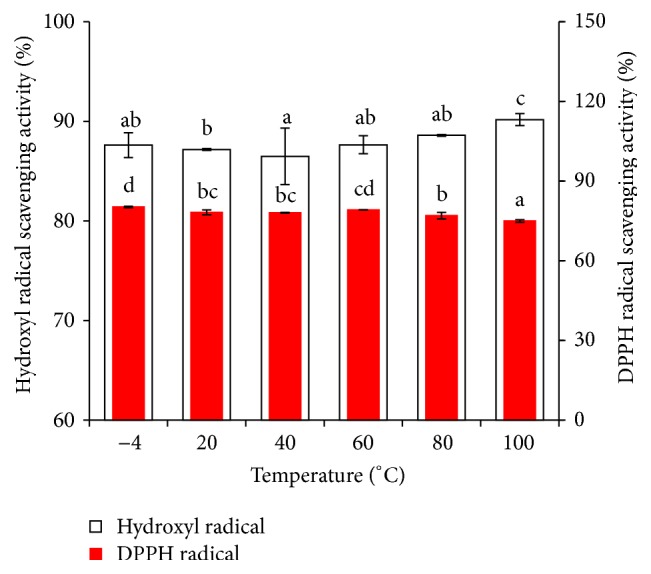
Effect of extraction temperature on antioxidant activity of MPHs. Different letters indicate significant differences between groups (*P* < 0.05).

Heat treatment is a general process in food manufacturing that can influence the functional properties of protein hydrolysates. During peptide processing, concentration and drying were examined, which are both processes related to temperature. As shown in Figure 5, the antioxidant activity of MPHs remained steady at temperatures of −4, 20, 40, and 60°C. When the temperatures were 80 and 100°C, the hydroxyl radical scavenging activity decreased slightly and DPPH radical scavenging activity increased slightly. Maybe the higher temperature leads to the protein denaturant and affects the antioxidant activity of MPHs. While the trends of hydroxyl radical scavenging activity and DPPH radical scavenging activity were different, we thought that there may be a little experiment error between them and need further research. In addition, Zhu et al. [20] have reported that the DPPH radical scavenging activity of peptides showed a sharp decline between 60 to 80°C, potentially due to the high temperature affecting the secondary structure, which could lead to the instability of antioxidant activity; then with the temperature continue increasing, the DPPH scavenging activity becomes stable. While in our experimental temperature range, hydroxyl radical scavenging activity of MPHs was maintained at 75–80.2% and the DPPH radical scavenging activity was maintained at 86.5–90.2%, respectively. This result indicated that the MPHs had good heat stability.

#### 3.4.2. Effect of pH on the Antioxidant Activity of MPHs

**Figure 6:**
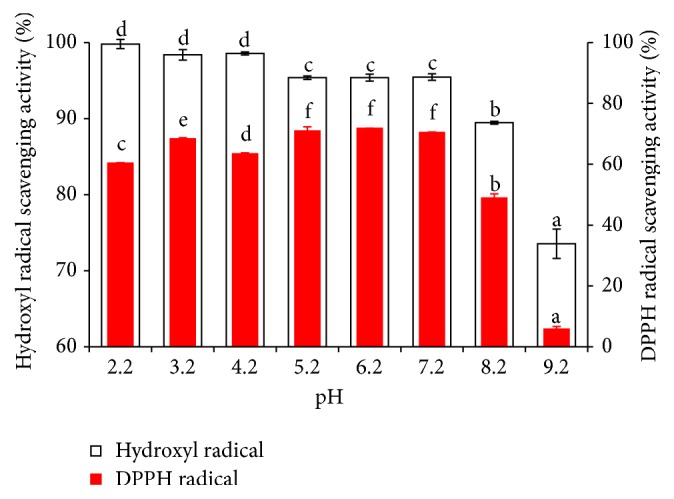
Effect of pH on antioxidant activity of MPHs. Different letters indicate significant differences between groups (*P* < 0.05).

The antioxidant activity of MPHs at different pH values was shown in Figure 6. At pH levels from 2.2 to 7.2, MPHs exhibited strong antioxidant activity. However, when the pH was 9.2, the DPPH and hydroxyl radical antioxidant activity of MPHs declined sharply, exhibiting reductions of 90% and 16%, respectively, compared with that under the pH of 2.2. Some researchers have found that when peptide is in alkaline condition, it is likely that racemization reaction occurs and reduces the antioxidant activity of MPHs; furthermore, at high pH values, deamination reaction resulting in change with structure, conformation, and loss of antioxidant activity of peptides might occur [40, 41]. Generally speaking, different peptides have different proper pH range, and they have high bioactivity during the pH range. Some other researchers have indicated that higher pHs, specially from 9.0 on, will promote the amino-group ionization from amino acids and peptides, increasing the H^+^ release and consequently enhancing the free radicals quenching, promoting the observed antioxidant activity [42]. In this section, the result showed that alkaline conditions were unfavorable for maintaining the antioxidant activity of MPHs.

#### 3.4.3. Effect of the Freeze-Thaw Cycle on the Antioxidant Activity of MPHs

**Figure 7:**
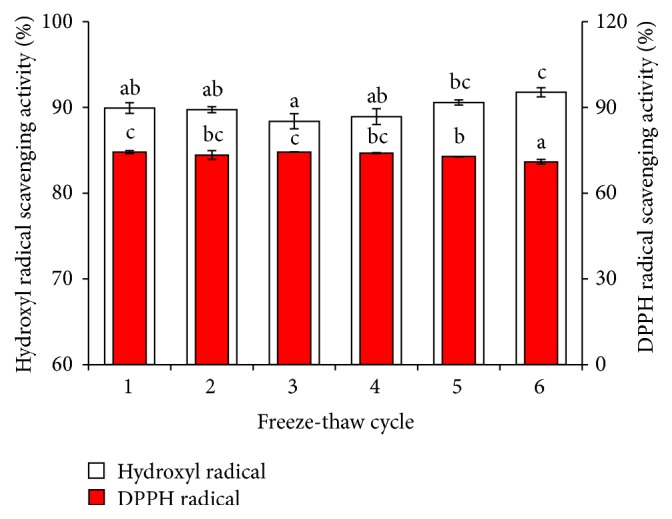
Effect of freeze-thaw cycle on antioxidant activity of MPHs. Different letters indicate significant differences between groups (*P* < 0.05).

During transportation and storage, high temperature, long hours, and enzyme degradation may influence seafood, so the freezing technology has been successfully applied, such that the frozen storage is an important preservation method for seafood. Thanonkaew et al. [43] have determined that lipid oxidation of all treatments increased as the number of freeze-thaw cycles increased. Maybe the protein or peptide degradation has reduced the antioxidant activity; on the other hand, structure and conformation of protein or peptide would change with rapid changes in temperature that might affect the antioxidant activity. However, in our study, we found that the DPPH scavenging activity was only reduced by 0.05% at the sixth freeze-thaw cycle in Figure 7, and hydroxyl radical scavenging activity also has little change with the freeze-thaw cycle increasing, which indicated that freeze-thaw cycles had little effect on the antioxidant activity of MPHs. We inferred that MPHs has high antioxidant activity stability along with the temperature shock, but we found white precipitation in the sample, likely from protein denaturation and precipitation caused by low temperatures. Therefore, although the result indicated that MPHs could be stored in low temperature conditions over multiple freeze-thaw cycles, we also should reduce the number of freeze-thaw cycles to avoid the generation of precipitation.

#### 3.4.4. Effect of Metal Ions on the Antioxidant Activity of MPHs

During food processing, metal ions are always preset in the ingredients used for food product preparation. Therefore, evaluation of their influence in food materials is essential. Thanonkaew et al. [43] examined the effect of different metal ions at various concentrations on lipid oxidation of muscle protein in cuttlefish (*Sepia pharaonis*), determining that Fe^2+^ induced lipid oxidation most effectively, and Cu^+^, Cu^2+^, and Cd^2+^ displayed negligible effects on lipid oxidation. Dawidowicz and Olszowy [44] have reported that iron and copper significantly influence the estimated antioxidant activity in an ABTS assay.

**Figure 8:**
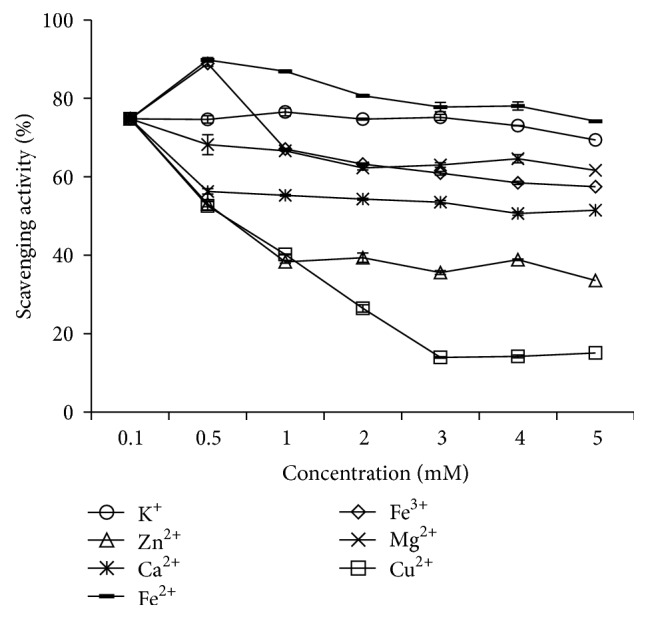
Effect of metal ion on DPPH radical scavenging activity of MPHs.

As shown in Figure 8, we found that all the tested metal ions had an effect on the DPPH radical scavenging activity of MPHs. Fe^2+^, K^+^, Mg^2+^, Ca^2+^, and Fe^3+^ had little effect on the DPPH scavenging activity of MPHs; for example, when the Fe^2+^ concentration increased from 0.1 mM to 5 mM, the DPPH antioxidant activity of MPHs was reduced from 74.8% to 57.5%. On the contrary, Zn^2+^ and Cu^2+^ notably reduced DPPH scavenging activity; for example, when the concentrations of Zn^2+^ and Cu^2+^ were 5 mM, the DPPH antioxidant activity of MPHs was reduced by 41.2% and 59.7%, respectively. This result indicated that Zn^2+^ and Cu^2+^ had a negative effect on the DPPH scavenging activity on MPHs. Dawidowicz and Olszowy [45] had indicated that the presence of metal ions in the measuring system blocks the scavenging process of DPPH radicals. Furthermore, Dawidowicz et al. [46] also studied that the increase of Cu^2+^ and Fe^3+^ concentration caused an almost linear deceleration of the DPPH^•^/antioxidant reaction kinetics, and the change of the reaction can be attributed to the formation of metal complexes with the components of measuring system.

Also, it is well known that hydroxyl radical is highly reactive and attacks proteins, DNA, and almost any biological molecule it touches. The damage may cause cancer, atherosclerosis, and neurodegenerative diseases [47]. Hence, the activity of scavenging hydroxyl radical is an important indicator for the antioxidant activity.

**Figure 9:**
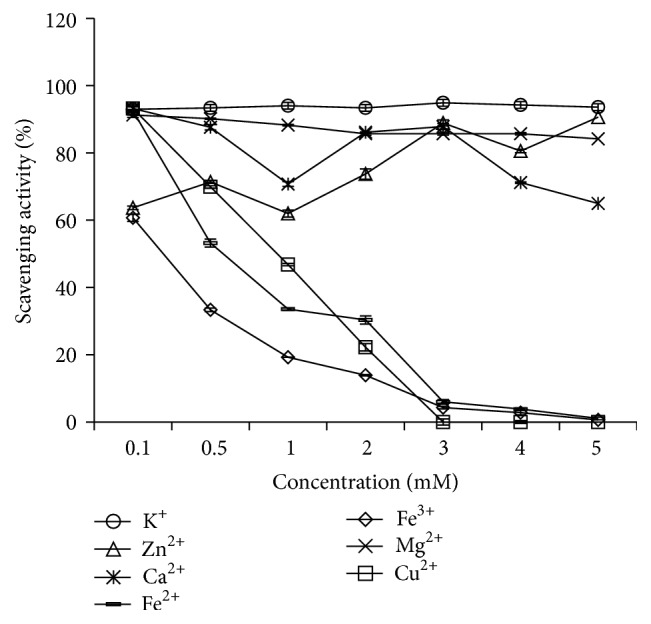
Effect of metal ion on hydroxyl radical scavenging activity of MPHs.

As shown in Figure 9, different metal ions had distinctly different effects on the hydroxyl radical scavenging activity of MPHs. K^+^, Zn^2+^, Ca^2+^, and Mg^2+^ had little effect on hydroxyl radical scavenging activity and they had no dose-dependent effect. However, Fe^2+^, Fe^3+^, and Cu^2+^ had a clear effect, especially at high concentrations, where the hydroxyl radical scavenging activity was almost lost; for example, when the concentrations of Fe^2+^, Fe^3+^, and Cu^2+^ were 5 mM, the hydroxyl radical antioxidant activity of MPHs was reduced by 91.5%, 60%, and 93.2%, respectively. It was probable that metal ions could catalyze H_2_O_2_ to produce more hydroxyl radicals, thus decreasing the hydroxyl radical scavenging activity of MPHs. This result showed that, in the MPH production process, the sample should avoid mixing with Fe^2+^, Fe^3+^, Zn^2+^, and Cu^2+^.

## 4. Conclusions

In this work, conditions of peptides extraction from mackerel protein were optimized by a CCD, and the optimum extraction conditions were as follows: enzyme concentration of 1726.85 U/g, pH of 7.00, temperature of 39.55°C, time of 5.5 h, and water/material ratio of 25 : 1. The highest yield of DPPH scavenging activity was at 79.14%. The 3D response surfaces showed that the enzyme concentration should control below 1800 U/g; the higher enzyme concentration was costly and could reduce the antioxidant activity. In addition, DPPH scavenging activity increased slightly when the extraction time increased from 4 to 5.5 h; this means that the DPPH scavenging activity was stable in 4 h later; then we should do more experiment about the extraction time below 4 h, because too long reaction time was consuming more energy in application. Furthermore, in our study, the water/material ratio was 25 : 1. The higher water/material ratio may decrease the concentration of the enzyme and affect the hydrolysis result, and a higher solvent volume will be outweighed by the difficultly of having to handle the extra volume of solvent. Besides, the antioxidant stability tests indicated that the MPHs were resistant to high temperature and not suitable for used in alkaline condition. Furthermore, the MPHs were sensitive to metal ions of Fe^2+^, Fe^3+^, Zn^2+^, and Cu^2+^, which were the essential trace element in the human body; further research should focus on reducing the influence of the metal ions mentioned above on MPHs.

## Figures and Tables

**Table 1:** Independent variables and their levels in CCD.

Variables	Code	Levels and range				
		−2	−1	0	1	2
Enzyme concentration (U/g)	*X*_1_	1200	1400	1600	1800	2000
pH	*X*_2_	5.5	6	6.5	7	7.5
Extraction temperature (°C)	*X*_3_	30	35	40	45	50
Extraction time (h)	*X*_4_	4	4.5	5	5.5	6
Water/material ratio (w/w)	*X*_5_	10	15	20	25	30

**Table 2:** Experimental design and result of response surface.

Run numbers	*X*_1_: enzyme concentration (U/g)	*X*_2_: pH	*X*_3_: extraction temperature (°C)	*X*_4_: extraction time (h)	*X*_5_: water/material ratio (w/w)	Response (%)
1	1400	6	35	4.5	15	57.84
2	1800	6	35	4.5	15	59.73
3	1400	7	35	4.5	15	58.38
4	1800	7	35	4.5	15	60.54
5	1400	6	45	4.5	15	62.97
6	1800	6	45	4.5	15	66.49
7	1400	7	45	4.5	15	65.14
8	1800	7	45	4.5	15	68.11
9	1400	6	35	5.5	15	53.51
10	1800	6	35	5.5	15	56.22
11	1400	7	35	5.5	15	55.68
12	1800	7	35	5.5	15	59.46
13	1400	6	45	5.5	15	61.08
14	1800	6	45	5.5	15	63.24
15	1400	7	45	5.5	15	64.59
16	1800	7	45	5.5	15	67.57
17	1400	6	35	4.5	25	64.59
18	1800	6	35	4.5	25	68.11
19	1400	7	35	4.5	25	69.73
20	1800	7	35	4.5	25	72.16
21	1400	6	45	4.5	25	69.46
22	1800	6	45	4.5	25	70.00
23	1400	7	45	4.5	25	70.81
24	1800	7	45	4.5	25	71.89
25	1400	6	35	5.5	25	69.19
26	1800	6	35	5.5	25	72.16
27	1400	7	35	5.5	25	72.43
28	1800	7	35	5.5	25	81.62
29	1400	6	45	5.5	25	71.35
30	1800	6	45	5.5	25	74.86
31	1400	7	45	5.5	25	72.16
32	1800	7	45	5.5	25	75.41
33	1200	6.5	40	5	20	63.51
34	2000	6.5	40	5	20	66.22
35	1600	5.5	40	5	20	71.35
36	1600	7.5	40	5	20	72.97
37	1600	6.5	30	5	20	61.89
38	1600	6.5	50	5	20	71.62
39	1600	6.5	40	4	20	65.68
40	1600	6.5	40	6	20	72.97
41	1600	6.5	40	5	10	52.97
42	1600	6.5	40	5	30	74.05
43	1600	6.5	40	5	20	74.59
44	1600	6.5	40	5	20	74.05
45	1600	6.5	40	5	20	71.35
46	1600	6.5	40	5	20	71.35
47	1600	6.5	40	5	20	70.81
48	1600	6.5	40	5	20	70.27
49	1600	6.5	40	5	20	70.81
50	1600	6.5	40	5	20	70.27

**Table 3:** ANOVA for response surface quadratic model.

Variables	Sum of squares	DF	Mean square	*F* value	*P* value
Model	1857.34	20	92.87	31.17	<0.0001
*X*_1_	73.05	1	73.05	24.52	<0.0001
*X*_2_	57.86	1	57.86	19.42	<0.0001
*X*_3_	173.24	1	173.24	58.15	<0.0001
*X*_4_	21.30	1	21.30	7.15	0.0122
*X*_5_	1077.11	1	1077.11	361.56	<0.0001
*X*_1_ *X*_2_	1.54	1	1.54	0.52	0.4775
*X*_1_ *X*_3_	2.34	1	2.34	0.78	0.3830
*X*_1_ *X*_4_	4.83	1	4.83	1.62	0.2130
*X*_1_ *X*_5_	0.58	1	0.58	0.20	0.6611
*X*_2_ *X*_3_	4.83	1	4.83	1.62	0.2130
*X*_2_ *X*_4_	2.96	1	2.96	0.99	0.3272
*X*_2_ *X*_5_	2.05	1	2.05	0.69	0.4131
*X*_3_ *X*_4_	0.45	1	0.45	0.15	0.7012
*X*_3_ *X*_5_	84.15	1	84.15	28.25	<0.0001
*X*_4_ *X*_5_	78.97	1	78.97	26.51	<0.0001
*X*_1_^2^	88.41	1	88.41	29.68	<0.0001
*X*_2_^2^	0.84	1	0.84	0.28	0.5991
*X*_3_^2^	45.25	1	45.25	15.19	0.0005
*X*_4_^2^	9.59	1	9.59	3.22	0.0833
*X*_5_^2^	128.00	1	128.00	42.97	<0.0001
Residual	86.39	29	2.98		
Lack of fit	66.56	22	3.03	1.07	0.5010
Pure error	19.83	7	2.83		
Cor. total	1943.74	49			
*R* ^2^	0.9556				
Adj. *R*^2^	0.9249				
Pred. *R*^2^	0.8591				
Adeq. precision	22.175				
CV%	2.56				
